# Experience-Induced Change of Alcohol-Related Risk Perception in Patients with Alcohol Use Disorders

**DOI:** 10.3389/fpsyg.2017.01967

**Published:** 2017-11-13

**Authors:** Sarah Klepper, Michael Odenwald, Susanne Rösner, Smeralda Senn, Hans Menning, Devi Pereyra-Kröll, Brigitte Rockstroh

**Affiliations:** ^1^Department of Psychology, University of Konstanz, Konstanz, Germany; ^2^Forel-Clinic, Ellikon, Zürich, Switzerland

**Keywords:** alcohol addiction, risk perception, risk reappraisal, treatment, feedback

## Abstract

The role of alcohol-related risk perception for effective treatment of alcohol use disorders (AUD) is still unclear. The present study on 101 alcohol-dependent patients undergoing a 10-week AUD treatment protocol investigated the relationship between alcohol-related risk perception and alcohol use with the hypotheses that (1) risk perception changes across treatment, (2) changes vary with treatment-related experiences of abstinence/relapse indicating ‘risk reappraisal,’ and (3) adjustment of perceived own vulnerability according to ‘risk reappraisal hypothesis’ predicts abstinence during follow-up. Abstinence during treatment was related to a decrease, and relapse during treatment to a slight increase in perceived own risks. Abstinence during the 3-month follow-up varied with experience-induced risk reappraisal. The results show an impact of risk reappraisal on alcohol use and hence advocate a focus on risk reappraisal in AUD treatment.

## Introduction

Most patients with alcohol use disorders (AUDs) know the deleterious consequences of harmful drinking (Arolt and Driessen, 1996; Room et al., 2005; Kraus et al., 2009). However, many patients fail to remain abstinent even after AUD treatment (Monahan and Finney, 1996; Miller et al., 2001). Distorted risk perception, like misjudged own vulnerability for negative consequences of alcohol use, has been discussed as a potential reason for the discrepancy between knowledge and behavior in patients with AUDs (Greenfield and Rogers, 1999; Miller and Rollnick, 2012). The interaction of risk perception and preventive behavior exhibited in a treatment setting as well as the impact of risk perception on alcohol use and treatment outcome is still unclear.

Research on risk perception in public health and illness prevention (Rosenstock et al., 1988; Renner and Schupp, 2005) evaluates risk perception along the four dimensions (Loewenstein et al., 2001; Borrelli et al., 2010): (1) perceived own vulnerability to experience negative consequences of a risky behavior (Gerrard et al., 1996); (2) chances of similar negative consequences experienced by a peer; (3) affective meaning of these consequences (Loewenstein et al., 2001); and (4) expected control over negative consequences by refraining from risk behavior (Bandura, 1976; Weinstein et al., 2008). These dimensions were conceptualized and documented for various risk domains including alcohol-related risks. For instance, a low perceived own vulnerability to experience negative consequences of alcohol use proved to be a risk factor for alcohol use in (non-addicted) adolescents (Lundborg and Lindgren, 2002; Substance Abuse and Mental Health Services Administration, 2009), and risky alcohol use varied with perceived own vulnerability in adults (Wild et al., 2001). In a previous study including patients with AUDs (Klepper et al., 2016), we found a positive relationship between alcohol use before AUD treatment and self-evaluated risk perception across all four dimensions.

Brewer et al. (2004) described the relationship between perceived own vulnerability and risk behavior in three ways : (1) perception of own risks ‘accurately’ reflects risk behavior, (2) high perceived vulnerability ‘motivates’ preventive behavior, and (3) preventive (or risky) behavior leads to ‘reappraisal’ of own vulnerability as higher/lower due to the respective behavior. All three hypotheses received empirical support: High perceived own vulnerability varying with high alcohol use in non-addicted adults (Sjöberg, 1998; Wild and Cunningham, 2001) and in AUD inpatients undergoing treatment (Klepper et al., 2016) support the ‘accuracy hypothesis’. Lasting reduced substance use consequent upon elevated risk perception (Grevenstein et al., 2015) is line with the ‘behavior motivation hypothesis.’ Finally, the ‘risk reappraisal hypothesis’ is supported by altered evaluation of own risks after cancer screening (in first-degree relatives of cancer patients; Glenn et al., 2011) and after vaccination (Brewer et al., 2007), thus, preventive behavior. While no studies so far included AUD patients, ‘risk reappraisal’ might be evident in changes of alcohol-related risk perception upon treatment success (abstinence functioning as preventive behavior). In turn, ‘risk reappraisal’ in the course of treatment should ‘motivate’ preventive behavior, i.e., abstinence following treatment.

The present study scrutinizes the relationship between alcohol-related risk perception and alcohol use in the context of AUD treatment: if risk perception influences alcohol use and treatment motivation, the study of treatment-mediated changes of alcohol-related risk perception (i.e., risk reappraisal) and of treatment outcome consequent upon risk reappraisal might improve the understanding of the discrepancy between risk knowledge and behavior and advance treatment.

In support of the ‘accuracy hypothesis,’ our previous comparison of patients with AUDs and control samples verified elevated self-ratings in patients relative to controls on all four dimensions of risk perception (Klepper et al., 2016). The present study involves a sample of patients with AUDs undergoing AUD treatment with the hypotheses: (1) that risk perception changes across treatment; (2) that changes in terms of ‘risk reappraisal hypothesis’ vary with treatment-related experiences (i.e., abstinence versus relapse during treatment); and (3) that patients who display ‘risk reappraisal’ – that is, perceived own vulnerability adjusted to (relapse/abstinence) experience during treatment – achieve abstinence during follow-up.

## Materials and Methods

### Participants

**FIGURE 1:**
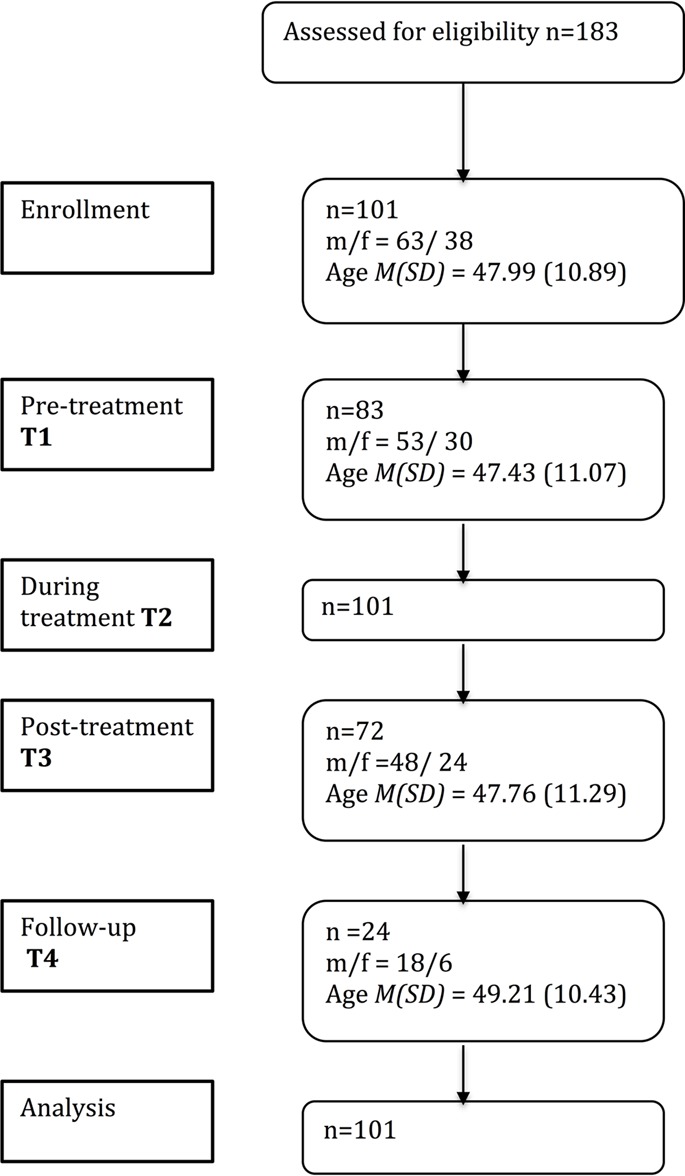
Flowchart of participants across different points of assessment. *N* = 62 participated in T1 and T3. T2 denotes the point in time, when information about alcohol use during treatment was available from patient files of all 101 participants, so that *n* = 101 data sets were submitted to hypotheses testing.

Patients with a diagnosis of alcohol dependence (ICD code F10) were recruited within a 10-week AUD treatment program at the Forel Clinic (Ellikon, Switzerland). *N* = 183 patients from those admitted to the Forel clinic during recruitment period were invited to participate. Exclusion criteria were suicidal tendencies, as well as insufficient language and intellectual capacity to complete the questionnaires. From this invited sample *n* = 101 agreed in participation as confirmed by written consent. As detailed in **Figure 1**, pre-treatment data of *n* = 83 and post-treatment data of *n* = 72 from *n* = 101 patients were available for data analyses. From this sample, *n* = 24 patients were reached 3 months after discharge (T4) for follow-up assessment via telephone. Reasons for drop-outs during treatment were: pre-mature cancelation, not interested in (continuing) participation, and concurrent therapy at testing date. Reasons for drop-outs after treatment were: change of telephone number, not available via telephone due to illness or death, relocation, and refusal to participate.

### Design and Procedure

The study protocol was approved by the Ethical Review Board of the Canton Zurich, Switzerland (Registration KEK-ZH.:2013-0594). Prior to the assessments, participants were informed about the purpose of the study and the assessment protocol and signed written informed consent according to the Declaration of Helsinki. Risk perception was assessed by means of a questionnaire prior to treatment onset (T1) and at program completion (T3). Recruitment and data collection extended from March 2014 to January 2015. Although data assessment was accomplished separately for each participant, assessments were arranged on the same day for groups of three to five patients, who started the AUD treatment program within the same week. Alcohol use was measured at T1 using the TLFB interview while alcohol use during treatment (T2) was gathered from clinic records. Follow-up assessment 3 months after discharge (T4) targeted those patients who completed treatment, were still available and agreed to participate. As patients undergoing treatment at the Forel Clinic came from all over Switzerland, follow-up screening for alcohol use was only possible by telephone interview, in which *n* = 24 were reached. The treatment program included weekly individual psychotherapy sessions, group psychotherapy, sports and physical exercises, occupational therapy and social counseling.

### Measures and Instruments

*Risk perception* was screened with the 20-item version of the German questionnaire “Fragebogen zur alkoholbezogenen Risikowahrnehmung” (FAR; Klepper et al., 2016; adequate test quality of the 20-item version was determined by item, reliability and validity analyses similar to the original 44-items version; Agricola, 2015), which captures alcohol-related risk perception by the four domains: (1) perceived own vulnerability (POV); (2) peer vulnerability (PV); (3) affective risk perception (AR); and (4) precaution effectiveness (PE). Five items per domain address typical alcohol-related problems and consequences in the fields of health, work and social life, as derived from reports in the literature. The domains are introduced as follows: POV: “How high do you rate your own risk if you maintain your drinking habits – of …?”, PV: “How high do you rate the risk of a peer in your age and gender with comparable drinking habits – of…?”, AR: “Imagine the following negative consequences as a result of your alcohol consumption. How threatening and worrying is the thought of …?”, PE: “If you permanently abstain from alcohol, how much would your personal risk of … decrease?”. Participants evaluated each item on a five-level Likert scale (POV and PV between 1 (very low) and 5 (very high), AR and PE between 1 (very little) and 5 (very strong).

*Alcohol use* in the month before admission (T1) and at follow-up (T4) were measured with the Timeline Followback interview (TLFB; Sobell and Sobell, 1992), a detailed diary of daily alcohol use. High test-retest reliability and validity are reported for the TLFB (Carey, 1997). ‘Total number of drinks’ (the sum of drinks converted into standard drinks) was used as a dependent variable.

Any alcohol use during the 10-week treatment period was considered as *relapse*, no alcohol use as *abstinence*. During treatment, alcohol use was not allowed, although risky situations, such as an overnight stay at home, were part of the treatment program. Alcohol use in such situations did not result in a premature interruption of treatment and would typically be used in individual and group therapy to improve the patients’ insight into their alcohol problems. Alcohol use was assessed by self-report, third-party information, standard breathalyzer and urine tests at the clinic, and was documented in the patients’ files. Alcohol use was assumed if any of the sources indicated drinking during treatment.

### Data Analysis

The change in risk perception by treatment (hypothesis 1) and the change in risk perception from pre- (T1) to post-treatment (T3) as a function of relapse/abstinence during treatment (hypothesis 2) were examined using a multivariate linear mixed model with the between-subject variable ‘group’ (relapse, abstinence) and ‘time’ (T1, T3). Linear mixed models allow intra-class correlations between assessments and control for missing data by estimating parameters from available data instead of deleting cases with missing data. The four FAR scales (POV, PV, AR, PE) were treated as dependent variables, while ‘time’ (T1, T3) and ‘group’ (relapse/abstinence) were included as predictors.

Following the definition of ‘risk reappraisal’ as a change in POV consequent upon preventive behavior, the impact of risk reappraisal on abstinence during follow-up (hypothesis 3) was evaluated for POV only. According to the ‘risk reappraisal hypothesis’ (Brewer et al., 2004), risk reappraisal is represented by a POV decrease consequent upon abstinence and a POV increase consequent upon relapse during treatment. A risk reappraisal score (RS) was determined as the product of changes in POV (x_2_ posttest score- x_1_ pretest score) and alcohol use (AU) during treatment; yes = “1” no = “-1”). The transformation (x_2_ – x_1_)^∗^AU reflects the amount of reappraisal by positive values for POV adjustment to relapse/abstinence and negative values for no POV change as a function of experience. A reliable increase in RS was determined according to the reliable change index (RCI) as described by Jacobson and Truax (1991). Cronbach’s Alpha of the POV scale (0.901; Klepper et al., 2016) was used to calculate RCI, as no test-retest reliability coefficient has yet been published. RCI can be interpreted analogous to a *z*-score – that is, for RCI > 1.96 the change is reliable on a 5% type-1 error level. According to RCI, RS ≥ 4 is considered to be a reliable pre-post change (alpha = 0.05). The relationship between reliable POV change (yes/no) and relapse/abstinence during follow-up was tested using Fisher’s Exact Test. As a proof of validity of the post-hoc RS score, a Receiver Operating Characteristic (ROC curve) was determined, illustrating the relationship between RS (predictor) and abstinence at follow-up (outcome).

## Results

**Figure 1** summarizes the sample sizes for the different assessments at T1, T3 and T4. Samples did not differ in gender distribution [χ^2^(3) = 1.49, *p* = 0.68], age [*F*(3,276) = 0.17, *p* = 0.92], and pre-treatment alcohol use [*F*(3,235) = 0.49 *p* = 0.69]. Little’s Missing Completely at Random (MCAR) Test revealed that missing questionnaire data were random (χ^2^= 337.63, *p* = 0.12; Little, 1988).

**FIGURE 2:**
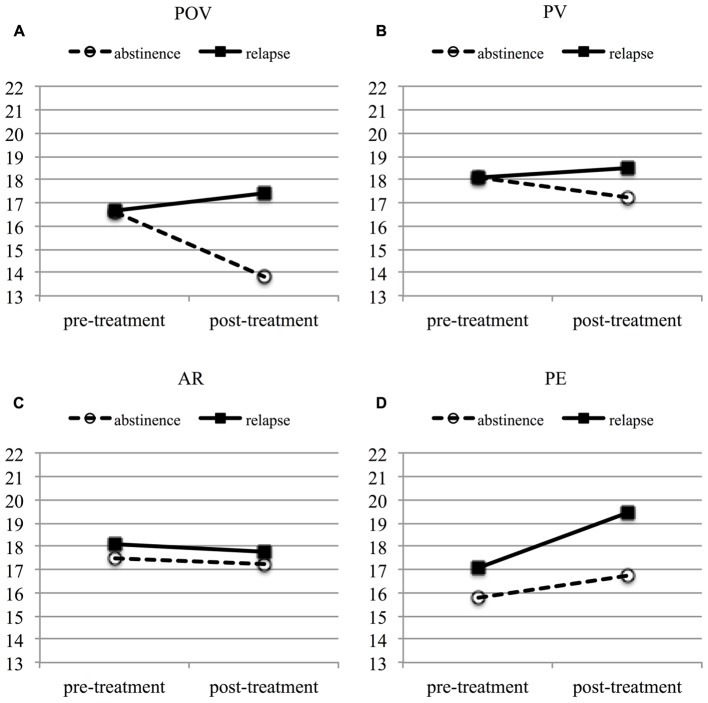
Linear functions of fixed predicted values (multivariate linear mixed model) to visualize the change in the FAR subscales **(A)** POV, **(B)** PV, **(C)** AR, and **(D)** PE over a treatment episode by relapse/abstinence. Solid line: patients who relapsed during treatment. Dashed line: patients who successfully abstained from alcohol during treatment. POV, perceived own vulnerability; PV, peer vulnerability; AR, affective risk perception; PE, precaution effectiveness.

The multivariate linear mixed model including the four FAR scales as dependent variables did not confirm an overall change in risk perception from pre- to post-treatment [time, *F*(4,80.41) = 1.62, *p* = 0.18], while post-hoc univariate mixed models verified trends for POV decrease [time, *F*(1,84.65) = 2.62, *p* = 0.11, *d* = -0.21; *M*_post_-*M*_pre_ = -1.01, *SE* = 0.63] and PE increase after treatment [time, *F*(1,85.91) = 3.41, *p* = 0.07, *d* = 0.25, *M*_post_-*M*_pre_ = 1.67, *SE* = 0.9]. However, the small overall changes in risk perception, when averaged across patients, resulted from an interaction of risk perception change (time) and experience during treatment (group) in the multivariate model [*F*(4, 80.41) = 2.87, *p* = 0.028]. In support of hypothesis (2), **Figure 2** illustrates a decrease in POV in those patients who abstained from drinking during treatment, and a slight increase in POV in those who relapsed during treatment (time ^∗^ group interaction, *F*(1,77.95) = 5.22, *p* = 0.025; for the simple effects time per group: abstinence (*n* = 58) *F* = 13.74, *p* < 0.001, *M*_post_-*M*_pre_ = -2.81, *SE* = 0.76), relapse (*n* = 43), *F* = 0.61, *p* = 0.44, *M*_post_-*M*_pre_ = 0.78, *SE* = 1). No comparable interaction could be confirmed for the other domains (*p* > 0.2). A main effect ‘group’ (*F* = 3.91, *p* = 0.05) verified higher POV scores in patients who relapsed compared to those who did not (*M*_relapse_-*M*_abstinence_ = 1.83, *SE* = 0.93).

Per hypothesis (3), risk reappraisal by experience during treatment (relapse/abstinence) was expected to modify treatment outcome as measured by relapse/abstinence at follow-up (T4). Among the 24 patients who participated in the follow-up assessment, *n* = 14 (58%) had consumed alcohol during follow-up, while 10 had remained abstinent. Risk reappraisal, defined as change in POV as a function of relapse/abstinence during treatment, was evident in *n* = 13 patients (per Reliable Change Index with critical pre-post difference ≥ 4 points). Relapse or abstinence during follow-up was significantly associated with a reliable reappraisal of perceived own vulnerability (*p* = 0.047), with 80% of abstinent participants showing reliable change according to RCI compared to 36% of those who relapsed. Moreover, the ROC curve confirmed the usefulness of the quantitative reappraisal score (RS) to predict relapse/abstinence during follow-up as indicated by a fair area under the curve (AUC = 0.761, *p* < 0.05, Youngstrom, 2014). A cut-off difference of >1.5 points identified the best compromise between perfect sensitivity (1.0, 100% correct positive) and fair specificity (0.64; 36% false positives). According to this cut-off difference, 100% of abstinent patients at follow-up showed risk reappraisal, which further supports risk reappraisal as marker of successful AUD treatment.

## Discussion

In health and addiction research risk perception, especially perceived own vulnerability to experience negative consequences of risk behavior has been linked to preventive behavior (Renner and Schupp, 2005; Brewer et al., 2007). The present study evaluates the relationship of alcohol-related risk perception and preventive behavior in patients with AUDs in the context of AUD treatment with different perspectives: the impact of treatment on risk perception, and particularly the impact of experiences of successful or failed behavior change during treatment (abstinence or relapse) on risk perception change and the impact of this risk reappraisal as a function of experiences during treatment on treatment outcome (i.e., post-treatment abstinence). Results emphasize risk perception as a function of experiences during treatment while overall changes in risk perception on the four dimensions across treatment period are small. Importantly, risk reappraisal (i.e., the adjustment of perceived own risk to experience negative consequences of risky alcohol use) is related to abstinence (i.e., health-promoting behavior upon treatment). Even though these results have to be substantiated and replicated for larger samples, they inform the role of risk perception for health-enhancing behavior and the understanding of the confusing discrepancy between risk knowledge and risky alcohol use: we propose that treatment-facilitated risk reappraisal is a crucial marker of treatment effects and should hence be key indicator for AUD treatment programs.

Risk perception was assessed on four dimensions, three addressing cognitive aspects of risk perception (e.g., perceived own vulnerability, peer vulnerability and precaution effectiveness) and one addressing the affective connotation of risk. In a previous study, patients with AUD had displayed higher scores than a healthy control group on all dimensions (Klepper et al., 2016), supporting Brewer’s ‘accuracy hypothesis’ (Brewer et al., 2004). In the present study, only perceived own vulnerability and expected benefit from precautious behavior proved sensitive to treatment and treatment-related experiences. The inverse relationship of reduced POV and increased PE after treatment is in line with evidence from other health domains (Alhakami and Slovic, 1994; Weinberger et al., 2010) on interrelated perceived own risks and expected benefits from behavioral change. AUD treatment programs might tackle this relationship when patients face risky situations such as overnight stays at home with uncontrolled access to alcohol, and therefore capitalize on the awareness of vulnerability together with the awareness of the benefits of behavior changes – as manifested in larger changes across treatment on these dimensions. In contrast, treatment does not influence the affective meaning of risks (AR). Affective connotation may vary with the type of risk; abuse-related risks challenge control and remain threatening, while AR of external risks such as nuclear power may be changed with information given (Slovic and Peters, 2006). Similarly, the evaluation of risks for comparable peers does not change with own treatment experiences.

The change in POV across treatment varies as a function of abstinence or relapse during the treatment period. This experience can be described as feedback on one’s own treatment success. Such feedback encourages and intensifies treatment motivation, while the negative feedback of relapse might either intensify efforts to succeed or dampen treatment motivation. Both effects may have guided the patient’s attention to risk evaluation and thereby contributed to the observed risk reappraisal. According to Renner et al. (2008), the decline in perceived risks consequent upon preventive behavior is crucial for ongoing motivation to maintain health-promoting behavioral change. In the case of patients with AUDs, elevated risk perception after relapse might also be crucial to maintain treatment motivation (Brewer et al., 2004). Our results might indicate that a basic ‘health strategy’ is preserved in patients with AUDs (Brewer et al., 2004; Renner et al., 2008). This alcohol-related risk reappraisal may have affected protective behavior, as a higher reliable reappraisal rate is evident in patients reporting abstinence after treatment. Experience-based reappraisal of own risks may be indicative of the ability to learn by experiences (success or failure), and this ability might mediate treatment effects in general. This hypothesis might be also addressed by studying cognitive and executive functions.

In cancer research, Glenn et al. (2011) report an impact of preventive behavior on risk perception in relatives of cancer patients similar to the impact of risk reappraisal on alcohol use during follow-up in the present study. However, the authors observe that group differences (preventive vs. non-preventive behavior) in risk perception diminished over time. Thus, diminishing risk perception after successful treatment might become a risk factor for a relapse over the long term (according to ‘behavior motivation hypothesis,’ Brewer et al., 2004). This dynamic change in POV might contribute to the frequent relapses in patients with AUDs. The finding of a decrease in POV after having performed health-promoting behavior also emphasizes the need to examine compensating behaviors (as in other addictions, comorbidities) after long-term abstinence and consequential changes in risk perception.

Limitations of the present study have to be noted. First of all, the small sample available for follow-up assessment and the short follow-up period render the present results preliminary. The present results need to be substantiated and replicated for a larger sample of patients with AUDs accomplishing the entire treatment and post-treatment period, and the latter should be longer. Secondly, risk perception was assessed by self-report, which can be biased through social desirability responding(Van de Mortel, 2008) and lack of respondence (Fox-Wasylyshyn and El-Masri, 2005). Thirdly, results might be biased because of high drop-out rate, as those participants might rather have dropped out or canceled treatment earlier with increased cognitive impairments (O’Leary et al., 1979) and/or a longer history of alcohol abuse; both variables could have influenced our measurement of risk perception. Fourthly, risk perception was only assessed at two time points. Further insights into the development and the effect of changes in risk perception are expected from longitudinal designs, including the influence of frequent relapses. Because the present study used conditional risk assessment for alcohol-specific risks (Halpern-Felsher et al., 2001), our risk perception measure can appropriately be linked to alcohol use. Through a longitudinal design with two points for measuring risk perception and three points for measuring alcohol relevant behavior (relapse during and after treatment), we were able to account for the change in risk perception and its interaction with behavior. Possible confounding variables such as therapeutic process and relevant experiences such as life events during treatment should be assessed.

Taken together, the results show risk reappraisal as a function of experiences (relapse/abstinence) during treatment and an impact of risk reappraisal on alcohol use, and therefore advocate for a focus on risk reappraisal in AUD treatment.

## Author Contributions

SK, MO, SR, SS, HM, DP-K, and BR have all contributed to the design, drafting, critical revision and final approval of the manuscript version. SK and MO conducted statistical analyses.

## Conflict of Interest Statement

The authors declare that the research was conducted in the absence of any commercial or financial relationships that could be construed as a potential conflict of interest.
